# Commentary: LAT1 expression in head and neck cancer: a prognostic biomarker with potential relevance for BNCT

**DOI:** 10.3389/fonc.2026.1779030

**Published:** 2026-02-27

**Authors:** Xiaobing Liao, Yuanyu Zhu, Changqian Piao, Yixin Zhang, Yong Tang

**Affiliations:** Changchun University of Chinese Medicine, Changchun, China

**Keywords:** boron neutron capture therapy (BNCT), head and neck squamous cell carcinoma (HNSCC), immunometabolism, LAT1 (SLC7A5), post-translational regulation, prognostic biomarker, theranostics, Tumor microenvironment (TME)

## 1 Introduction

L-type amino acid transporter 1 (LAT1), encoded by the SLC7A5 gene, is a transmembrane protein essential for the uptake of large neutral amino acids, such as leucine and phenylalanine, which support anabolic tumor growth. In a recent study published in Frontiers in Oncology, the prognostic significance of LAT1 was investigated by Cavalieri et al. (1) in a cohort of 100 patients with locally advanced head and neck squamous cell carcinoma (HNSCC). High LAT1 mRNA expression was reported as an independent predictor of poor overall survival (OS). Furthermore, this expression was found to be significantly enriched in “hypoxic” and “mesenchymal” transcriptomic clusters. Conversely, the lowest LAT1 expression was observed in the “immune-reactive” cluster.

These findings are particularly relevant to Boron Neutron Capture Therapy (BNCT). BNCT is a binary radiotherapy reliant on the selective accumulation of boron-10 within tumor cells. Given that the boron carrier boronophenylalanine (BPA) serves as a specific substrate for LAT1, this study provides a compelling biological rationale for the application of BNCT in radioresistant HNSCC characterized by high LAT1 expression. However, bridging the gap between transcriptomic profiling and clinical BNCT efficacy requires addressing critical translational challenges, specifically the correlation between mRNA and functional protein levels, as well as the immunometabolic consequences of LAT1 overexpression.Addressing these gaps is essential to validate LAT1 not only as a functional biomarker for BNCT candidate selection but also as a pivotal regulator of the tumor immunometabolic environment.

## 2 From transcriptome to functional phenotype

SLC7A5 levels were quantified by Cavalieri et al. using Affymetrix ClariomD chips, establishing a robust association between gene expression and aggressive molecular subtypes. While transcriptomics offers high-throughput screening capabilities, it serves as a surrogate marker rather than a direct measure of transporter function. LAT1 does not function autonomously; it requires the heavy chain glycoprotein CD98 (SLC3A2) to form a heterodimeric complex, which stabilizes the transporter at the plasma membrane (2).

Although a correlation between SLC7A5 and SLC3A2 mRNA was observed, cell surface expression may be significantly altered by post-translational regulation. Specifically, the functional landscape of LAT1 is shaped by three key regulatory nodes:

Ubiquitylation and Degradation: Under metabolic stress, the E3 ubiquitin ligase Nedd4-2 (NEDD4L) targets lysine residues (K19, K25, K30) on the LAT1 N-terminus (3), triggered by TXNIP as an adaptor protein (4). This modification recruits the ESCRT complex (notably ESCRT-0/Hrs) to sort LAT1 into multivesicular bodies for lysosomal degradation (5).

Vesicular Trafficking: The dynamic recycling of LAT1 is governed by Rab-GTPases. While Rab1 controls the anterograde transport from the ER to the Golgi, Rab11 maintains a sub-membranous “reserve pool” in the endocytic recycling compartment (ERC), allowing tumor cells to rapidly upregulate surface LAT1 density upon growth signal stimulation (6).

Membrane Environment and Chaperone Stability: The stability of the transporter is contingent upon the glycosylation state of its partner, CD98, and the cholesterol content of the plasma membrane. Cholesterol-rich lipid rafts provide the necessary conformational support for the heterodimer; notably, the depletion of membrane cholesterol has been shown to decrease the Vmax of LAT1, thereby reducing the rate of substrate uptake regardless of mRNA abundance (7).

Previous studies in lung and breast cancers have indicated that cytoplasmic retention or a lack of membrane localization—driven by these mechanisms—can render LAT1 non-functional for amino acid or drug uptake (8). Consequently, elevated mRNA levels do not automatically guarantee sufficient BPA accumulation for effective BNCT. For clinical translation, functional stratification remains paramount. While transcriptomics serves as an excellent screening tool, molecular imaging using LAT1-specific PET tracers, such as [18F]-FBPA, currently represents the most direct method for evaluating potential BNCT benefit. However, the quantitative thresholds and clinical correlation of [18F]-FBPA specifically within HNSCC cohorts are still under exploration; unlike its more established role in other malignancies such as gliomas, it has not yet been adopted as a routine clinical screening standard for HNSCC BNCT trials (9). Refining these imaging protocols is essential to ensure that a transcriptomic ‘high-LAT1’ profile accurately translates into therapeutic boron concentrations in a clinical setting. While functional stratification confirms LAT1’s role as a theranostic biomarker, its clinical impact extends beyond boron transport to the active modulation of nutrient availability within the head and neck cancer microenvironment.

## 3 The immunometabolic landscape

A striking observation in the original article is the inverse relationship between LAT1 expression and the immune-reactive phenotype. Gene clusters enriched for immune defense responses were found to exhibit the lowest LAT1 levels. This finding aligns with the concept of “metabolic competition” within the tumor microenvironment (TME). LAT1 is essential not only for tumor cells but also for T lymphocytes. Upon antigen recognition, T cells undergo metabolic reprogramming, upregulating Myc and subsequently SLC7A5 to support rapid clonal expansion (10).

In the nutrient-deprived TME, LAT1-overexpressing tumor cells are postulated to act as “metabolic parasites,” potentially depleting extracellular pools of essential amino acids. This depletion is hypothesized to trigger the Amino Acid Starvation Response (AASR) in infiltrating T cells, suppressing mTORC1 signaling and driving T cell anergy or exhaustion (11). However, while these interactions have been demonstrated primarily *in vitro* and in murine models, direct evidence within the human HNSCC microenvironment remains limited, and the link between transcriptomic clusters and active *in vivo* amino acid depletion remains speculative. Thus, the poor prognosis associated with high LAT1 expression in the cohort studied by Cavalieri et al. (1) may stem not only from intrinsic tumor radioresistance but also from potential LAT1-mediated immune suppression. This creates a “cold” immune microenvironment, consistent with the low LAT1 expression observed in the immune-reactive cluster. This metabolic competition underscores LAT1’s role as an immunometabolic regulator, providing a biological rationale for integrating metabolic targeting with particle radiotherapy.

## 4 Discussion and conclusion

The study by Cavalieri et al. (1) represents a significant step toward optimizing patient selection for Boron Neutron Capture Therapy (BNCT) in head and neck oncology. By identifying the enrichment of LAT1 in hypoxic and mesenchymal clusters—subtypes traditionally resistant to conventional photon radiotherapy—the authors highlight a specific niche where BNCT could provide maximum benefit. The high linear energy transfer (LET) of alpha particles generated in BNCT is less dependent on tissue oxygenation, offering a mechanistic advantage in these hypoxic, LAT1-high tumors (12).

However, several limitations in the original study warrant consideration to ensure scientific rigor. As a retrospective, single-institution analysis involving 100 patients, the generalizability of these findings to broader clinical settings remains to be confirmed. Furthermore, LAT1 expression was assessed solely at the transcriptomic level without corresponding protein-level validation. While the transcriptomic 6-cluster model utilized is biologically relevant, its stability and reproducibility across independent HNSCC cohorts require further prospective verification to establish it as a reliable stratification tool.

Future clinical designs should consider LAT1 as both a target for BNCT and a modulator of immunity. If high LAT1 expression indeed fosters an immunosuppressive environment via nutrient depletion, targeting these cells with BNCT may offer a dual benefit: direct debulking of radioresistant clones and the potential restoration of metabolic conditions favorable for immune infiltration (13). Nonetheless, the translation of this rational combination into clinical practice must be approached with caution. Currently, there are no published preclinical models specifically evaluating the synergy of BNCT and anti-PD-1/PD-L1 therapies in HNSCC, leaving the optimal sequencing and timing of such interventions entirely unexplored. For instance, while BNCT-induced immunogenic cell death could theoretically prime the immune response, the potential for immune-mediated bystander damage or exacerbated tissue toxicity from high-LET particles must be rigorously evaluated.

In conclusion, while transcriptomic validation provides a robust biological foundation, the integration of functional imaging and immunometabolic profiling will be essential to fully realize the potential of LAT1 as a theranostic biomarker in HNSCC. By recognizing LAT1 as both a gateway for BNCT and a master regulator of tumor immunity, we can develop more precise and synergetic therapeutic strategies for radioresistant head and neck cancers.
